# Runx1 contributes to articular cartilage maintenance by enhancement of cartilage matrix production and suppression of hypertrophic differentiation

**DOI:** 10.1038/s41598-019-43948-3

**Published:** 2019-05-21

**Authors:** Fumiko Yano, Shinsuke Ohba, Yasutaka Murahashi, Sakae Tanaka, Taku Saito, Ung-il Chung

**Affiliations:** 1 Bone and Cartilage Regenerative Medicine, Graduate School of Medicine, The University of Tokyo, Tokyo, Japan; 2 Center for Disease Biology and Integrative Medicine, Graduate School of Medicine, The University of Tokyo, Tokyo, Japan; 3 Sensory and Motor System Medicine, Graduate School of Medicine, The University of Tokyo, Tokyo, Japan; 4 Department of Bioengineering, Graduate School of Engineering, The University of Tokyo, Tokyo, Japan

**Keywords:** Cartilage development, Disease model

## Abstract

Osteoarthritis (OA) results from an imbalance of the dynamic equilibrium between the breakdown and repair of joint tissues. Previously, we reported that Runx1 enhanced chondrogenic differentiation through transcriptional induction of *COL2A1*, and suppressed hypertrophic differentiation. Here, we investigated the involvement of Runx1 in OA development as well as its potential underlying molecular mechanism. When we analysed OA development in *Col2a1-Cre;Runx1*^*fl/fl*^ and *Runx1*^*fl/fl*^ mice by surgically inducing joint instability, Cartilage degradation and osteophyte formation of *Col2a1-Cre;Runx1*^*fl/fl*^ joints was accelerated compared with joints in *Runx1*^*fl/fl*^ animals 8 weeks after surgery. To investigate chondrocyte regulation by Runx1, we analysed interactions with co-factors and downstream molecules. Runx1 enhanced cartilage matrix production in cooperation with Sox5, Sox6, and Sox9, and co-immunoprecipitation assays showed protein–protein binding between Runx1 and each Sox protein. Knockdown of Runx1 increased expression of a hypertrophic marker, Co10a1, in mouse articular cartilage and primary chondrocytes. This expression was accompanied by decreased expression of Bapx1, a potent suppressor of hypertrophic differentiation. Notably, Runx1-induced suppression of hypertrophic differentiation was diminished by siRNA silencing of *Bapx1*, whereas chondrogenic markers were unaltered. Thus, Runx1 contributes to articular cartilage maintenance by enhancing matrix production in cooperation with Sox proteins, and suppressing hypertrophic differentiation at least partly via Bapx1 induction.

## Introduction

Development of osteoarthritis (OA) – the most common degenerative joint disorder – results not only from an imbalance in the dynamic equilibrium between breakdown and repair of joint tissues, but also ectopic endochondral ossification under mechanical loading conditions^1,2^. During this process, chondrocytes undergo hypertrophic differentiation characterized by secretion of type X collagen (Col10a1). Next, avascular cartilage tissue is converted into highly vascularized bone tissue via degradation of cartilage matrix and vascular invasion^1,3^.

Runt-related transcription factor (Runx) family proteins, including Runx1, Runx2, and Runx3, play crucial roles in skeletal development^4,5^. In particular, Runx2 has been well characterized in skeletal development and OA. *Runx2* deletion results in a lack of ossification that impairs chondrocyte maturation^6^. Runx2 protein, which is highly expressed in the prehypertrophic and hypertrophic zones of limb epiphyseal cartilage, promotes hypertrophic differentiation^5,7^. Previously, we demonstrated suppression of OA development by *Runx2* haploinsufficiency^8^, which was recently confirmed using a chondrocyte-specific *Runx2* knockout^9^. Indeed, a series of studies concluded that *Runx2* deficiency decelerates OA development by suppressing hypertrophic differentiation^10^.

In contrast to Runx2, Runx1 is involved in early chondrogenic differentiation. Runx1, which is widely expressed by chondrocyte progenitors and stimulates chondrogenesis^4,11^. Previously, we reported that Runx1 enhanced cartilage matrix production and induced chondrogenic transcription factors such as sex determining region Y-box (Sox) genes^12,13^. Mechanistically, Runx1 activates the *COL2A1* promoter through specific binding to a Runx motif in the 5′-flanking region^12^. In addition, Runx1 suppresses hypertrophic differentiation of cultured chondrocytes^12^. In articular cartilage, *RUNX1* expression is downregulated in patients with OA compared with healthy individuals^12^. Mechanical compression induces upregulation of Runx1 in cartilage tissues, which contributes to chondrocyte proliferation^14^. Chondrogenic compounds, such as TD-198946 and Kartogenin, have been shown to function via Runx1 induction^12,15^. Moreover, we recently showed that intraarticular injection of polyplex nanomicelles containing *RUNX1* mRNA suppressed development of surgically-induced OA in mice^16^. Collectively, these data support a protective role of Runx1 with regard to articular cartilage maintenance; however, molecular mechanisms underlying enhancement of cartilage matrix production and suppression of hypertrophic differentiation by Runx1 are not well understood.

Herein, we investigated roles of Runx1 during OA development using chondrocyte-specific *Runx1* knockout mice. We further examined interactions between Runx1 and other chondrogenic factors in enhancement of cartilage matrix production, as well as the function of molecules downstream of Runx1 in regulation of hypertrophic differentiation.

## Results

### Runx1 deficiency enhanced OA development

**Figure 1:**
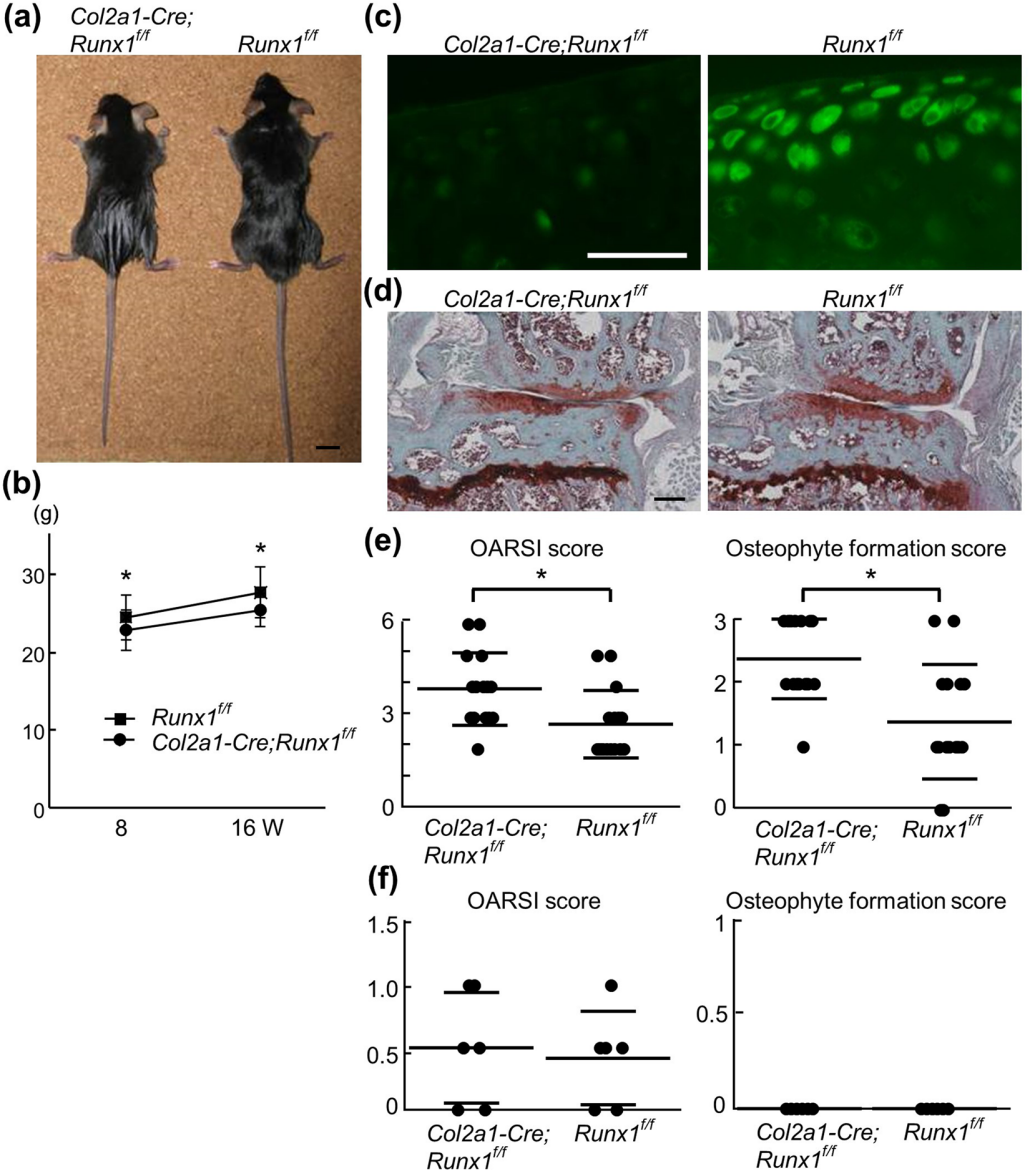
OA development in *Col2a1-Cre;Runx1*^*fl/fl*^ and *Runx1*^*fl/fl*^ mice. (**a**) Gross appearance of *Col2a1-Cre;Runx1*^*fl/fl*^ and *Runx1*^*fl/fl*^ littermates at 8 weeks of age. Scale bars, 10 mm. (**b**) Total body weight of *Col2a1-Cre;Runx1*^*fl/fl*^ and *Runx1*^*fl/fl*^ littermates at 8 or 16 weeks of age. Data are expressed as means (symbols) ± SD (error bars) of 15 mice per group. (**c**) Runx1 immunofluorescence in normal knee cartilage of *Col2a1-Cre;Runx1*^*fl/fl*^ and *Runx1*^*fl/fl*^ littermates at 16 weeks of age. Scale bars, 50 μm. (**d**) Safranin O staining of knee joints 8 weeks after OA surgery in *Col2a1-Cre;Runx1*^*fl/fl*^ and *Runx1*^*fl/fl*^ littermates. Scale bars, 200 μm. (**e**) Quantification of OA development by Osteoarthritis Research Society International (OARSI) grading system and osteophyte formation score. Data are expressed as means ± SD of 15 mice per group. *P < 0.05 vs. *Runx1*^*fl/fl*^. (**f**) Quantification of OA development by Osteoarthritis Research Society International (OARSI) grading system and osteophyte formation score in normal knee cartilage of *Col2a1-Cre;Runx1*^*fl/fl*^ and *Runx1*^*fl/fl*^ littermates at 16 weeks of age. Data are expressed as means ± SD of 6 mice per group.

**Figure 2:**
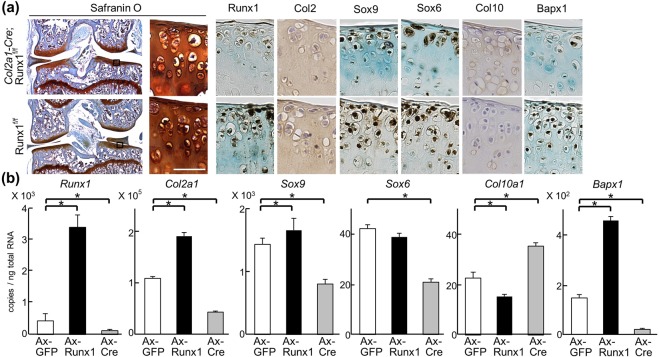
Altered marker expression by Runx1. (**a**) Safranin O staining and immunohistochemistry with antibodies to marker proteins in articular cartilage of 16-week-old *Col2a1-Cre;Runx1*^*fl/fl*^ and *Runx1*^*fl/fl*^ littermates under physiological conditions. Inset boxes in Safranin O staining indicate regions shown in enlarged safranin O and immunostaining images. Scale bars, 50 μm. (**b**) mRNA levels of marker genes in *Runx1*^*fl/fl*^ primary articular chondrocytes adenovirally transduced (Ax) with GFP, Runx1, or Cre after 5 days of culture. *P < 0.01 versus Ax-GFP. Data are expressed as mean ± SD of six samples per group.

First, the involvement of Runx1 in OA development was examined. Although no abnormalities were found in skeletal morphology or patterning, chondrocyte-specific *Runx1* knockout mice (*Col2a1-Cre;Runx1*^*fl/fl*^), showed slight dwarfism compared with their *Runx1*^*fl/fl*^ littermates at 8 weeks of age (Fig. 1a). Moreover, body weights of *Col2a1-Cre;Runx1*^*fl/fl*^ mice were about 10% less than that of control littermates throughout the experimental period (Fig. 1b). After confirming the efficient deletion of *Runx1* in adult articular chondrocytes (Fig. 1c), we created the surgical OA model^17^. Cartilage degradation and osteophyte formation of *Col2a1-Cre;Runx1*^*fl/fl*^ joints were significantly accelerated compared with *Runx1*^*fl/fl*^ littermate joints after 8 weeks, in spite of the significantly lighter body weight of *Col2a1-Cre;Runx1*^*fl/fl*^ mice (Fig. 1d,e). In contrast, there was no significant difference in OA progression between 16-week-old *Col2a1-Cre;Runx1*^*fl/fll*^ and *Runx1*^*fl/fl*^ littermates (Fig. 1f and see also Safranin-O staining in Fig. 2a). These data suggested that Runx1 can protect articular cartilages from OA-inducing stimuli.

### Runx1 deficiency induces downregulation of chondrogenic markers and upregulation of hypertrophic markers

Expression of marker proteins was examined in *Col2a1-Cre;Runx1*^*fl/fl*^ and *Runx1*^*fl/fl*^ cartilage under physiological conditions without any operation. Chondrogenic factors such as Sox6 and Sox9 were decreased in *Col2a1-Cre;Runx1*^*fl/fl*^ cartilage (Fig. 2a) as well as the expression of Runx1. In contrast, the hypertrophic marker Col10 was increased by Runx1 deletion (Fig. 2a and Supplementary Fig. S1a,b). Bapx1, a suppressive factor of chondrocyte hypertrophy, was clearly decreased in *Col2a1-Cre;Runx1*^*fl/fl*^ cartilage (Fig. 2a). We next quantified changes in mRNA levels of marker genes induced by Runx1 overexpression or knockdown. We obtained primary articular chondrocytes from *Runx1*^*fl/fl*^ mice, and transduced adenoviral GFP, Runx1, or Cre. *Col2a1*, *Sox9* and* Bapx1* were upregulated by Runx1 overexpression, while *Sox6* was unchanged (Fig. 2b). In contrast, *Runx1* deletion decreased all these markers (Fig. 2a,b). *Col10a1* were downregulated by Runx1 overexpression, and upregulated by Runx1 knockdown (Fig. 2b). These data are comparable to the result of immunohistochemistry. Thus, the series of *in vivo* and *in vitro* data supports negative roles of Runx1 in chondrocyte hypertrophy as well as positive ones in early chondrogenic differentiation in articular cartilages.

### Runx1 cooperates with Sox proteins to enhance cartilage matrix production

**Figure 3:**
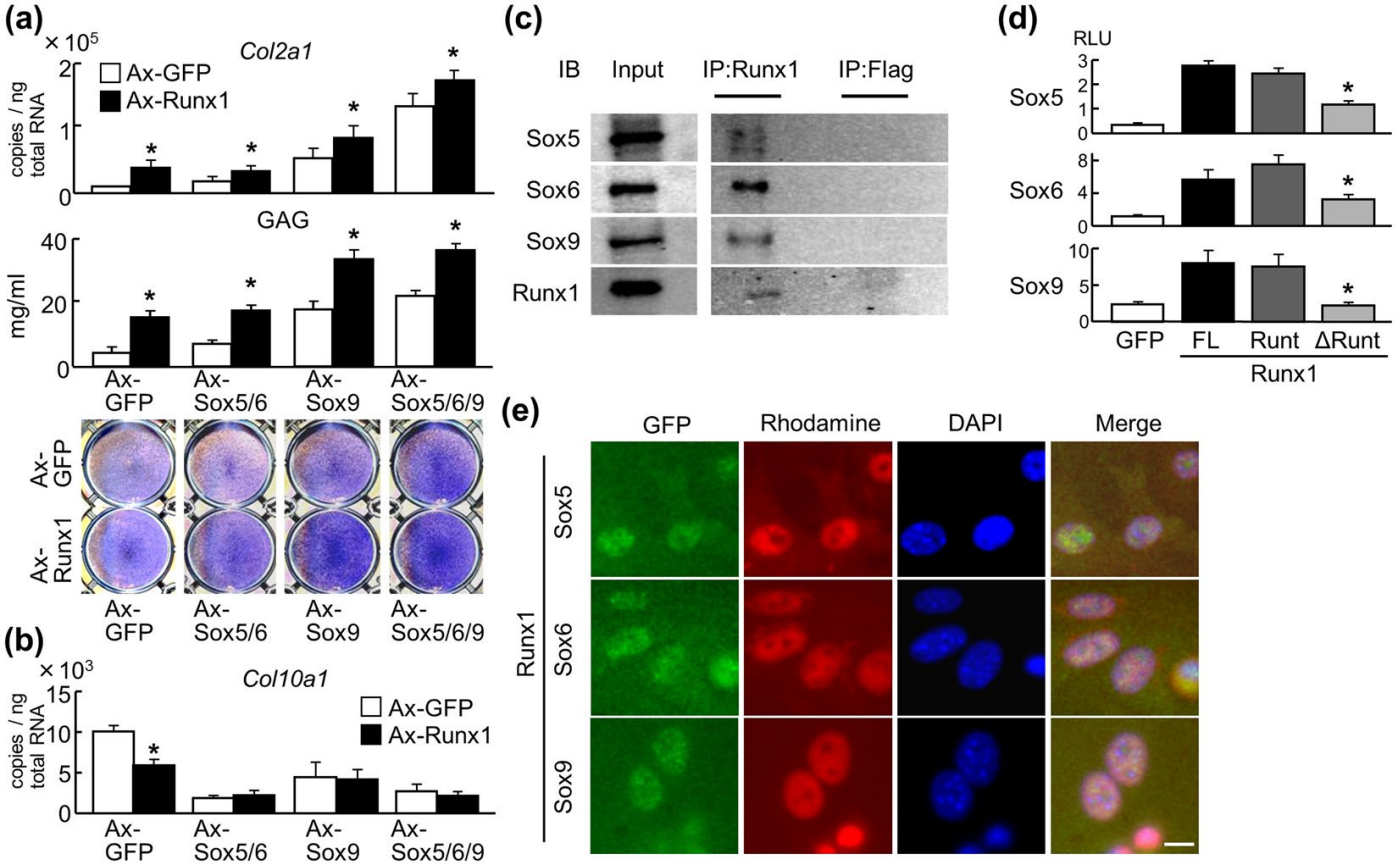
Interaction between Runx1 and Sox proteins. (**a**) Levels of *Col2a1* mRNA, glycosaminoglycan (GAG), and Toluidine blue staining in C3H10T1/2 cells adenovirally transduced with Runx1 in combination with GFP, Sox5 + Sox6, Sox9, or Sox5 + Sox6 + Sox9. Data are expressed as means (bars) ± SD (error bars) of six samples per group *P < 0.01 versus each Ax-GFP control. (**b**) Levels of *Col10a1* mRNA in ATDC5 cells adenovirally transduced with Runx1 in combination with GFP, Sox5 + Sox6, Sox9, or Sox5 + Sox6 + Sox9. Cells were cultured in pellets for 3 days after adenoviral transduction. Data are expressed as means (bars) ± SD (error bars) of four samples per group *P < 0.01 versus each Ax-GFP control. (**c**) Co-immunoprecipitation (Co-IP) assay using cell lysates of human articular chondrocytes. Cell lysates underwent IP with an antibody to Flag or Runx1, and were then immunoblotted with the other antibody. Full-length blots are presented in Supplementary Figure 2. (**d**) Mammalian two-hybrid assay by transfection of luciferase reporter vectors expressing GAL4–Runx1 (FL, full-length Runx1; Runt, the runt domain only; and ΔRunt, Runx1 mutant lacking the runt domain) and VP16–Sox proteins with GAL4 binding sites into HuH-7 cells. Data are expressed as means (bars) ± s.d. (error bars) of four samples per group *P < 0.01 vs. FL. (**e**) Immunocytochemistry of endogenous Runx1, Sox5, Sox6, and Sox9 in human articular chondrocytes were detected by secondary antibodies with red and green fluorescence, respectively. Scale bar, 10 μm.

Next, we examined interactions between Runx1 and Sox proteins. Adenoviral overexpression of Runx1 and Sox was initially performed in C3H10T1/2 cells, which were examined for chondrogenic differentiation. Levels of *Col2a1* mRNA, glycosaminoglycan (GAG), and Toluidine blue staining were increased by Runx1 alone, and further enhanced by co-overexpression of Sox5, Sox6, or Sox9 (Fig. 3a), indicating that Runx1 enhances cartilage matrix production in cooperation with Sox proteins. Upon transduction of these factors into ATDC5 cells and subsequent pellet culture to induce hypertrophic differentiation, *Col10a1* expression was significantly decreased by Runx1 or Sox overexpression; however, it was not further suppressed by co-overexpression (Fig. 3b), indicating suppression of hypertrophic differentiation by Runx1 is not associated with Sox proteins. We next investigated protein–protein interactions between Runx1 and Sox proteins. Co-IP using human articular chondrocytes showed specific binding between endogenous Runx1 and Sox5, Sox6, or Sox9 (Fig. 3c and Supplementary Fig. S2); the interaction was also confirmed by Co-IP in HEK293 cells overexpressing those proteins (Supplementary Fig. S3). A mammalian two-hybrid assay confirmed physical interactions between these proteins, and experiments using *Runx1* deletion mutants showed that the runt domain is essential for interactions with Sox proteins (Fig. 3d and Supplementary Fig. S4). Immunocytochemistry showed nuclear co-localization of endogenous Runx1 and Sox proteins (Fig. 3e). Immunohistochemistry for Sox and Runx proteins in mouse E17.5 tibial limb cartilage showed that Runx1 and Sox proteins were co-localized in periarticular and prehypertrophic chondrocytes. In contrast, Runx2 was primarily expressed in late differentiating chondrocytes in the hypertrophic zone (Supplementary Fig. S5). Taken together, these data suggest that Runx1 enhances cartilage matrix production partially through interactions with Sox5, Sox6, and Sox9.

### Co-localization and induction of Bapx1 by Runx1 in articular cartilage and meniscus

**Figure 4:**
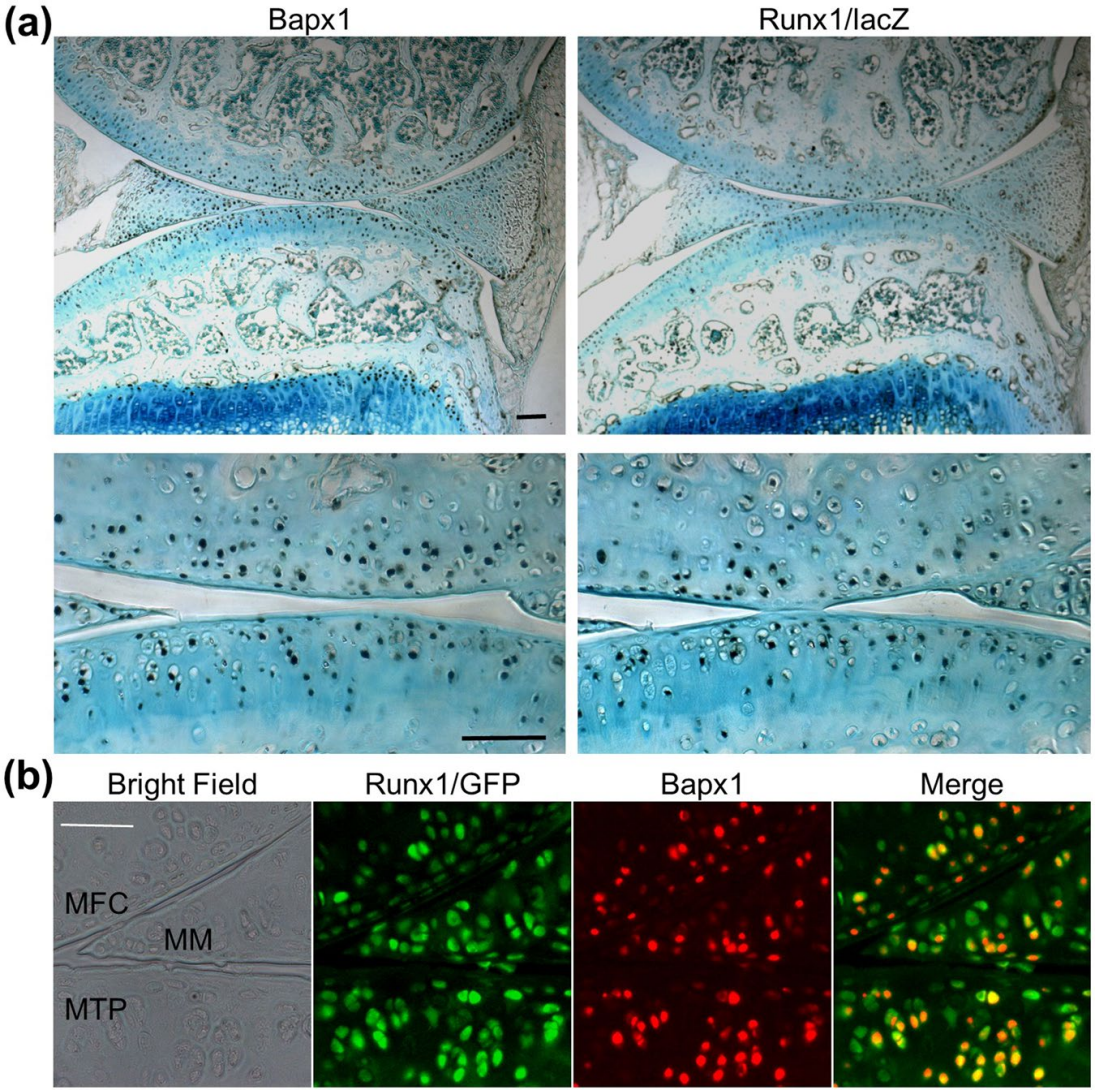
Expression patterns of Runx1 and Bapx1 in articular cartilage. (**a**) Immunohistochemistry with antibodies to Bapx1 and β-gal in articular cartilage of Runx1 heterozygous lacZ knock-in mice (*Runx*^*lz*/+^) under physiological conditions. Scale bars, 100 μm for low and high magnification images. (**b**) Immunofluorescence with antibodies to GFP and Bapx1 in knee joints of *Runx1-IRES-GFP* mice under physiological condition. Scale bar, 50 μm. MFC, medial femoral condyle; MTP, medial tibial plateau; MM, medial meniscus.

We then examined molecular mechanisms underlying suppression of hypertrophic differentiation by Runx1. Among candidate molecules demonstrating suppressive effects against hypertrophy, we focused on the transcription factor Bapx1, also known as Nkx3.2. Bapx1 is indispensable for skeletal development, as its deficiency leads to the impairment of hypertrophic differentiation and maturation of growth plate chondrocytes^18^. Bapx1 expression was markedly decreased in *Col2a1-Cre;Runx1*^*fl/fl*^ cartilage (Fig. 2a). In primary chondrocyte cultures, Bapx1 was increased by Runx1 overexpression, and decreased by Runx1 knockdown (Fig. 2b). Immunohistochemistry showed that Bapx1 protein was widely localized throughout normal articular cartilage and menisci of *Runx1* heterozygous knock-in mice (*Runx1*^*lz*/+^)^11^, similar to the distribution pattern of Runx1 (Fig. 4a). Indeed, upon performing co-immunofluorescence of Bapx1 and Runx1 in knee joints of *Runx1-IRES-GFP* knock-in mice^19^, Runx1 was mostly detected in Bapx1 expressed cells (Fig. 4b and Supplementary Fig. S6).

### Bapx1 mediates the suppression of hypertrophic differentiation by Runx1

**Figure 5:**
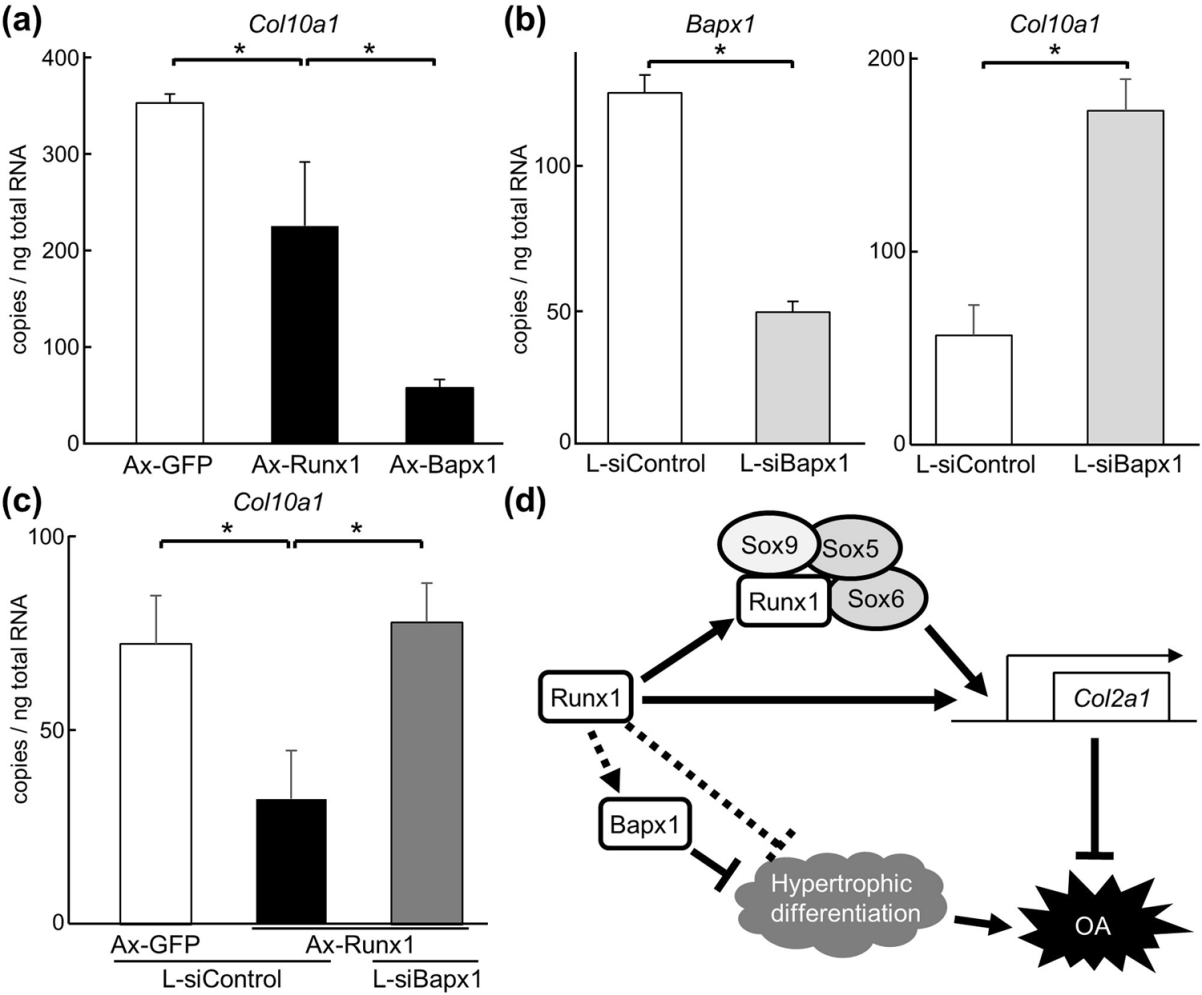
Regulation of chondrocyte hypertrophic differentiation by Runx1 and Bapx1. (**a**) Levels of *Col10a1* mRNA levels in primary chondrocytes adenovirally transfected with GFP, Runx1 or Bapx1 after 5 days of culture. Data are expressed as means (bars) ± s.d. (error bars) of three samples per group *P < 0.01. (**b**) Levels of *Bapx1* and *Col10a1* mRNA in primary chondrocytes lentivirally transfected with shRNAs against Bapx1(L-*siBapx1*) or control (L-siControl). Data are expressed as means (bars) ± s.d. (error bars) of three samples per group *P < 0.01. (**c**) Levels of *Col10a1* mRNA in primary articular chondrocytes adenovirally transfected with GFP or Runx1, and lentivirally transfected with L-*siBapx1* or L-siControl. Data are expressed as means (bars) ± SD (error bars) of three samples per group *P < 0.01. (**d**) Schematic of proposed mechanism for inhibitory effects of Runx1 against OA development. Runx1 induces *Col2a1* expression via direct binding to its 5′-flanking region and in cooperation with Sox5, Sox6, and Sox9. Runx1 suppresses hypertrophic differentiation through induction of Bapx1.

Finally, we investigated the association of Runx1 and Bapx1 in regulation of hypertrophy. When equal amounts of adenoviral vectors for Runx1 and Bapx1 were transduced into mouse primary chondrocytes, decreases in *Col10a1* induced by Bapx1 were more substantial than those induced by Runx1 (Fig. 5a). To examine Bapx1 loss-of-function, lentiviral transduction of siRNA was used to prepare Bapx1-downregulated chondrocytes. Although suppression of Bapx1 was partial, *Col10a1* was increased by Bapx1 suppression (Fig. 5b). Upon examining overexpression of Runx1 in Bapx1-downregulated and control cells, decreases of *Col10a1* induced by Runx1 were cancelled by Bapx1 suppression (Fig. 5c). These data suggest that Bapx1 at least partly mediates suppression of hypertrophic differentiation induced by Runx1.

## Discussion

The present study showed, using both *in vivo* and *in vitro* experiments, that Runx1 plays an essential role in articular cartilage maintenance. Chondrocyte-specific deletion of *Runx1* enhanced OA development, although the body weight of knockout mice was also less than that of control littermates. In *Runx1*-knockout cartilage, expression of Col2 and chondrogenic Sox proteins was decreased, while hypertrophic makers were increased. *In vitro* experiments revealed that Runx1 enhanced cartilage matrix production in cooperation with Sox5, Sox6, and Sox9. Meanwhile, Runx1 suppressed hypertrophic differentiation of chondrocytes through induction of Bapx1.

During endochondral ossification, Sox5, Sox6, and Sox9 are bona fide transcription factors that are indispensable for chondrogenesis^20^. Sox9, which is expressed in mesenchymal chondroprogenitor cells, broadly regulates chondrocyte differentiation and cartilage matrix production in conjunction with subsequently expressed Sox5 and Sox6^20,21^. Runx1 is also expressed in mesenchymal chondroprogenitor cells and limb chondrocytes at an early stage of differentiation^4^. Loss of Runx1 function results in impaired skeletal growth^22^; however, underlying mechanisms are not fully understood. The present data show that Runx1 cooperates with Sox5, Sox6, and Sox9 (through protein–protein binding via the runt domain of Runx1) to enhance cartilage matrix production. Although we did not experimentally examine this feature, it may also bind to a high-mobility-group (HMG)-type DNA-binding domain present in Sox proteins, as the HMG domain mediates protein–protein interactions^23^. Meanwhile, we previously identified the responsive element of Runx1 in the 5′-flanking region of the *Col2a1* promoter^12^. Considering that core Sox9 enhancers are localized in the first intron of the *Col2a1* gene^24^, Runx1 may enhance *Col2a1* transcription both directly and indirectly (Fig. 5d) to exert anabolic effects in chondrocytes of the developing skeleton and articular cartilage.

In the present study, we identified Bapx1 as a novel downstream molecule of Runx1. Bapx1-deficient mice exhibited lethal skeletal dysplasia, with abnormal development of the vertebral column^18,25^. Although the sclerotome cells of mutants appeared to migrate and condense normally into vertebral bodies, they failed to differentiate into hypertrophic chondrocytes^18^. Subsequent studies revealed that suppression of hypertrophic differentiation by Bapx1 is mediated by Runx2^26,27^. Additionally, Bapx1 contributes to chondrocyte viability through Rela activation^28^. A recent study using human primary articular chondrocytes showed that Bapx1 suppresses hypertrophic marker genes including *RUNX2*, *COL10A1*, and alkaline phosphatase, while Bapx1 did not influence expression of the chondrogenic factors *SOX9*, *COL2A1*, or aggrecan^29^. These reports support a suppressive function of Bapx1 on chondrocyte hypertrophy, although some others suggest opposite ones^18,25^. Considering these findings and the present data, Runx1 possibly suppresses hypertrophic chondrocyte differentiation in a Bapx1-dependent manner, and also suppresses subsequent OA development. However, involvement of the Runx1-Bapx1 pathway in skeletal development has yet to be examined; we are still unable to rule out the possibility that Runx1 and Bapx1 independently act on chondrocyte hypertrophy (Fig. 5d).

In conclusion, we demonstrated that Runx1 regulates articular cartilage by enhancing matrix production and suppressing hypertrophic differentiation via Bapx1 induction and/or another distinct pathway that induces some regulators for hypertrophy (Fig. 5d). These data indicate that Runx1 is a potent factor for the maintenance of articular joints, similar to previous studies demonstrating that TD-198946 and Kartogenin exert chondroprotective effects through Runx1 induction^12,15,30^. Thus, the results of the present study may contribute to the elucidation of molecular mechanisms underlying articular cartilage homeostasis and OA development.

## Methods

### Ethics statement

We performed all animal experiments according to a protocol approved by the animal care and use committee of the University of Tokyo. We obtained samples of human articular cartilage from three individuals undergoing total knee arthroplasty with written informed consent, as approved by the Ethics Committee of the University of Tokyo. All methods were performed in accordance with the relevant guidelines and regulations.

### Mice

In each experiment, we compared genotypes of littermates maintained in a C57BL/6 background with a standard diet. *Col2a1-Cre* mice and *Runx1-flox* mice were provided by Dr. Shu Takeda (Tokyo Medical and Dental University, Tokyo, Japan)^13^. To generate *Col2a1-Cre;Runx1*^*fl/fl*^ mice, *Runx1*^*fl/fl*^ mice were mated with *Col2a1*-Cre mice to obtain *Col2a1*-Cre;*Runx1*^*fl*/+^ mice, which were then mated with *Runx1*^*fl/fl*^ mice. Runx1 heterozygous LacZ knock-in mice were provided by Dr. Stefano Stifani (McGill University)^11^. *Runx1-IRES-GFP* mice were provided by Dr. James Downing (St Jude Children’s Research Hospital)^19^.

### OA experiment

The experimental OA model was performed on 8-week-old *Col2a1*-Cre;*Runx1*^fl/fl^ and *Runx1*^fl/fl^ littermate male mice, as previously described^17^. Briefly, under general anaesthesia, the medial collateral ligament was transected and the medial meniscus was removed using a surgical microscope. A sham operation was performed on the contralateral knee joint using the same approach, with no ligament transection or meniscectomy. All mice had their left knee joints operated on, while the right knees were subjected to sham operation. Mice were analysed 8 weeks after surgery. OA development was assessed by two blinded independent observers using the Osteoarthritis Research Society International (OARSI) scoring system^31^ and osteophyte formation score^17^.

### Biochemical measurement of glycosaminoglycan (GAG)

We collected whole cell lysates from C3H10T1/2 cells using an M-PER kit (Pierce Chemical), and evaluated GAG contents using and Alcian blue-binding assay (Wieslab).

### Co-immunoprecipitation (Co-IP) and mammalian two-hybrid assays

We collected whole cell lysates from human articular chondrocytes using an M-PER kit, and performed co-IP using a Catch and Release kit (Upstate Biotechnology, Lake Placid, NY) with anti-Flag (F7425, Sigma-Aldrich, St. Louis, MO), anti-Runx1 (23980), anti-Sox5 (94396), anti-Sox6 (30455) and anti-Sox9 (185230) antibodies (Abcam, Cambridge, UK). Immune complexes were eluted and subjected to SDS-PAGE. Mammalian two-hybrid assays were performed with the Checkmate Mammalian Two-Hybrid System (Promega, Madison, WI).

### Histological analyses

Sections were stained with Safranin O-fast green using standard protocols^32^. For immunohistochemistry, sections were incubated with antibodies to Runx1 (Abcam23980), Sox6 (Abcam30455), Sox9 (Abcam185230), Col10 (Affymetrix 14-9771-80; eBioscience, Austria), Col2 (Millipore MAB8887), GFP (Abcam 290,), Bapx1 (Abcam 83288) and β-gal (Promega Z378A) diluted at 1:500 by blocking reagent, and detected with a CSAII Biotin-free Tyramide Signal Amplication System (Dako, Glostrup, Denmark). For Col10 and Col2 sections were treated with hyaluronidase [25 mg/ml in phosphate-buffered saline (PBS)] for 30 min. Sections were counterstained with methyl green or hematoxylin.

### Immunocytochemistry

Human articular chondrocytes were fixed in 4% paraformaldehyde/PBS for 10 min and incubated for one hour with primary antibodies to Runx1 (mouse monoclonal, Abcam189172), Sox5 (Abcam94396), Sox6 (Abcam30455) or Sox9 (Abcam185230), which was diluted at 1:250 in blocking reagent, at room temperature. A secondary antibody conjugated with Alexa Fluor 488(anti-mouse) for Runx1 and 568 (anti-rabbit) (Molecular Probes, Eugene, OR) for Sox5, Sox6 and Sox9 were used as secondary antibodies, and the nucleus was counterstained with Hoechst 33258 (Sigma-Aldrich). Double immunofluorescence was visualized using Rabbit IgG Labeling Kit Zenon Alexa Fluor 488 (Z25302, Invitrogen, Carlsbad, CA) for detection of GFP, and Rabbit IgG Labeling Kit Zenon Alexa Fluor 647 (Z25308, Invitrogen) for detection of Bapx1.

### Cell cultures

We obtained samples of human articular cartilage from three individuals undergoing total knee arthroplasty after obtaining written informed consent from the patients. Human articular chondrocytes were isolated and cultured as previously described^33^. C3H10T1/2, HuH-7and ATDC5 cells (Riken BRC, Tokyo, Japan) were maintained in monolayer culture as previously described^34^. Primary mouse costal chondrocytes were isolated and the pellet cell cultures were performed. In addition, primary mouse articular chondrocytes were isolated and cultured as previously described^35^ for use in functional analyses. Adenovirus vectors for GFP, Runx1, Bapx1 and Cre were prepared as previously described^12^. Cells were transduced with adenoviral vectors at a multiplicity of infection (MOI) of 100. Lentivirus vectors of control shRNA (pSMART Non-targeting mCMV-TurboRFP) and *Bapx1* shRNA (pSMART 2.0 mCMV/turboRFP Nkx3.2) were purchased from Dharmacon (Lafayette, CO).

### Real-time RT-PCR analysis

Total RNA was extracted from cells using an RNeasy Mini kit (Qiagen, Hilden, Germany). One microgram of RNA was reverse-transcribed with a QuantiTect Reverse Transcription kit (Qiagen) to produce single-stranded cDNA. Real-time RT-PCR was performed with an ABI Prism 7500 Sequence Detection System (Applied Biosystems, Foster City, CA) using FastStart Universal SYBR Green Master Mix (Roche, Tokyo, Japan) with rodent actin as the internal control. Primer sequences are shown in Supplementary Table 1.

### Statistical analysis

Quantitative data were expressed as mean ± standard deviation (SD), with statistical significance evaluated using analysis of variance, Student t test or Mann-Whitney’s U Test for OARSI score, as appropriate. *P* values less than 0.05 were considered significant.

## Supplementary information

Supplementary Information
