# Supplementary material for: Runx1 contributes to articular cartilage maintenance by enhancement of cartilage matrix production and suppression of hypertrophic differentiation
Source: Sci Rep. 2019 May 21;9:7666. doi: 10.1038/s41598-019-43948-3 (PMC6529519; doi:10.1038/s41598-019-43948-3)
Supplement: Supplementary file 1 — Supplementary Information [file 41598_2019_43948_MOESM1_ESM.pdf]

Supplementary Figures and Table

Runx1 contributes to articular cartilage maintenance by enhancement of cartilage matrix production and suppression of hypertrophic differentiation

Fumiko Yano[1,2]*, Shinsuke Ohba[2], Yasutaka Murahashi[3], Sakae Tanaka[3], Taku Saito[3] and Ung-il Chung[2, 4]

[1] Bone and Cartilage Regenerative Medicine, [2] Center for Disease Biology and Integrative Medicine, [3] Sensory and Motor System Medicine, Faculty of Medicine, [4] Department of Bioengineering, The University of Tokyo, Tokyo, Japan.

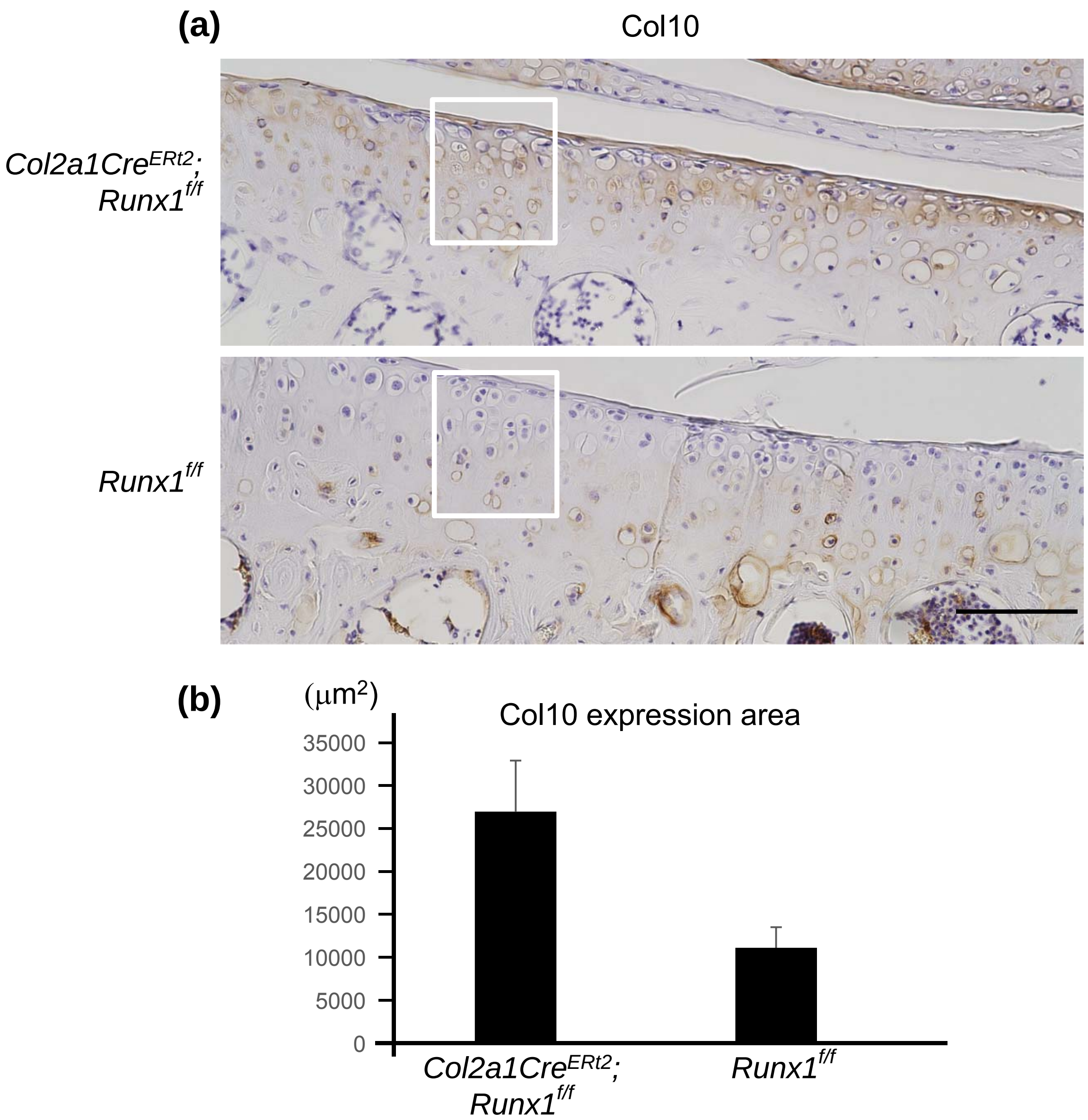

Supplementary Figure 1. (a) Immunohistochemistry of Col10 in articular cartilage of 16-week-old Col2a1-Cre;Runx1fl/fl and Runx1fl/fl littermates under physiological conditions. Inset boxes indicate regions shown in Figure 2A. Scale bars, 100 μm **(b)** The Col10 expression area in immunohistochemistry.

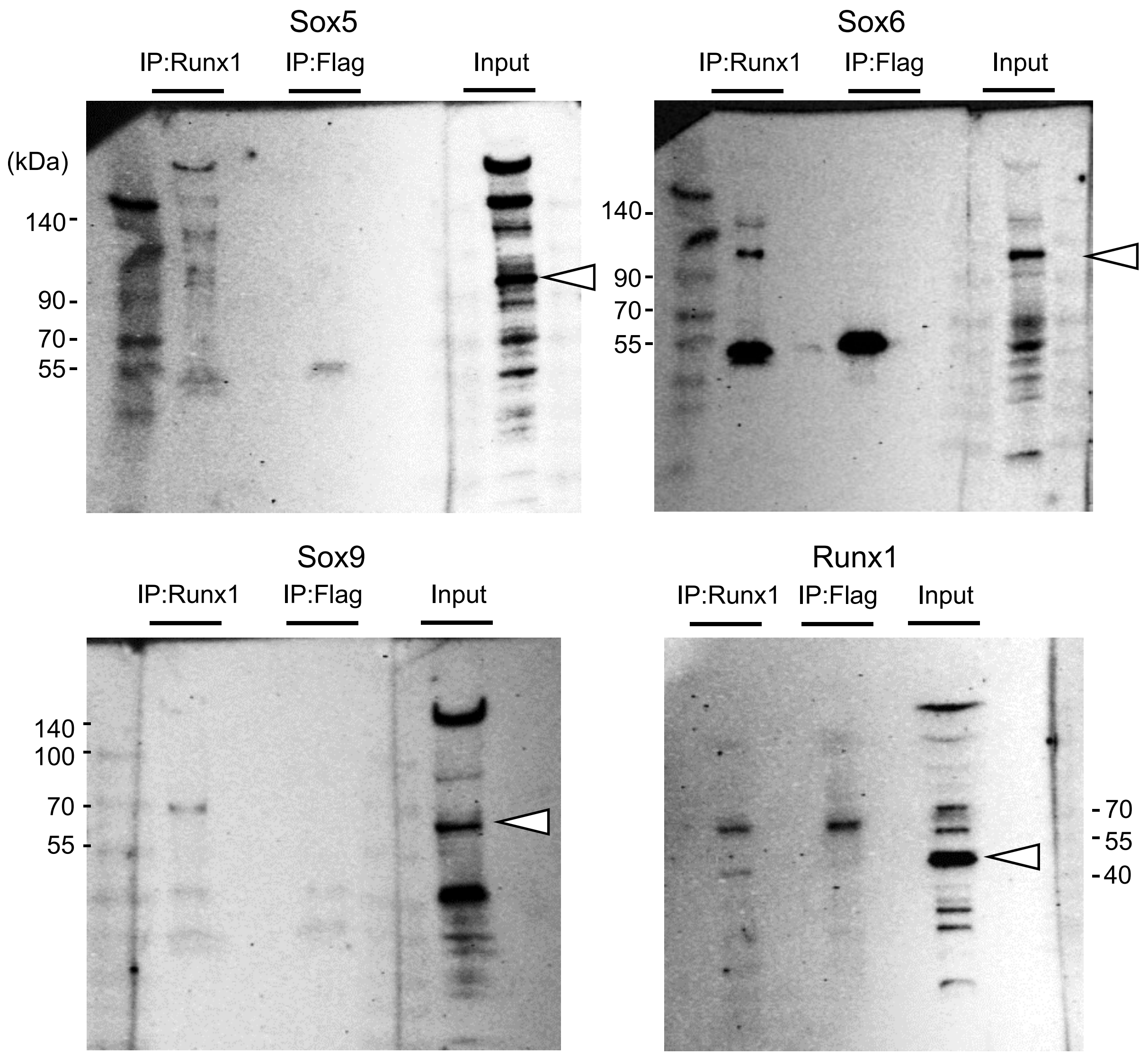

Supplementary Figure 2. Co-immunoprecipitation (Co-IP) assay using cell lysates of human articular chondrocytes. Cell lysates underwent IP with an antibody to Flag or Runx1, and were then immunoblotted with Sox5, Sox6 or Sox9. Arrowheads, detected Input protein. These images are full-length blot in Figure 3 (c)

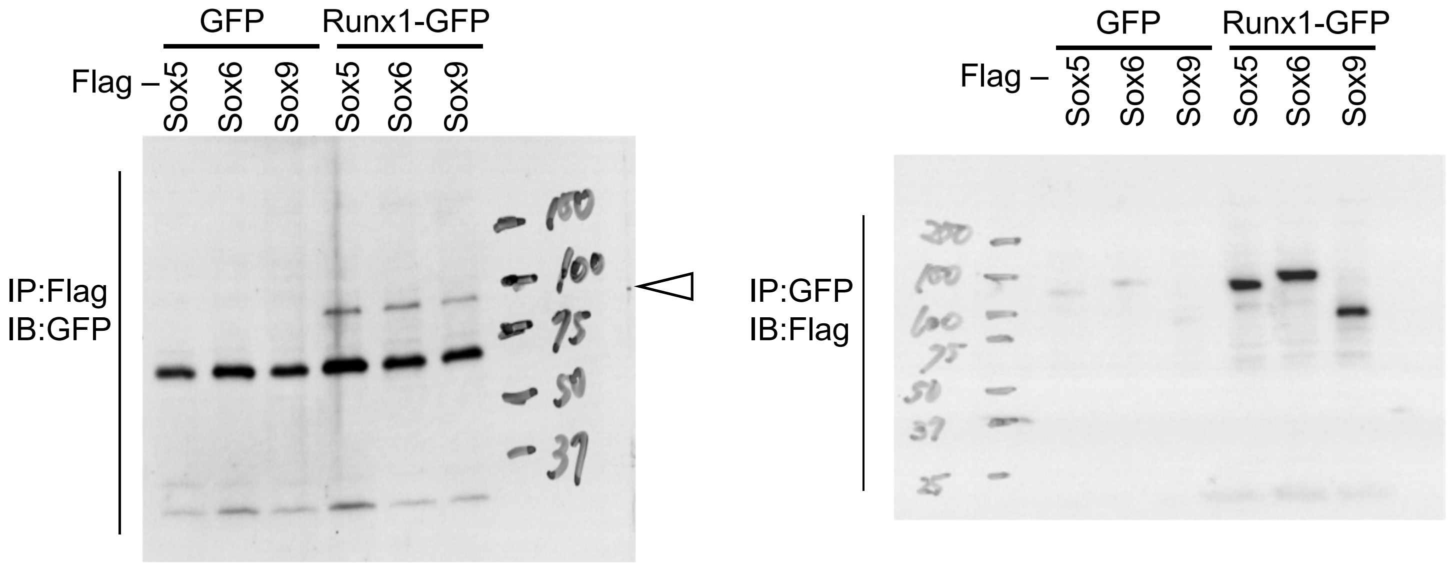

Supplementary Figure 3. Co-immunoprecipitation (Co-IP) assay using cell lysates of HEK293 transfected with GFP-tagged Runx1 and Flag-tagged Sox members. Cell lysates underwent IP with an antibody to Flag or GFP, and were then immunoblotted with the other antibody. Arrowheads, Runx1-GFP.

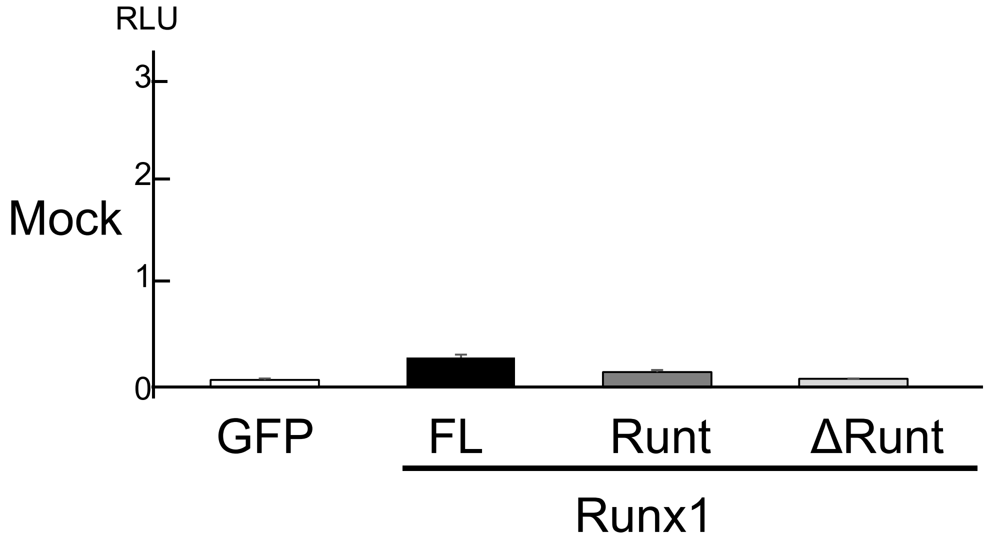

Supplementary Figure 4. Mammalian two-hybrid assay by transfection of luciferase reporter vectors expressing GAL4–Runx1 (FL, full-length Runx1; Runt, the runt domain only; and ΔRunt, Runx1 mutant lacking the runt domain) and VP16–Mock vector proteins as negative control with GAL4 binding sites into HuH-7 cells. Data are expressed as means (bars) $\pm$ s.d. (error bars) of four samples per group.

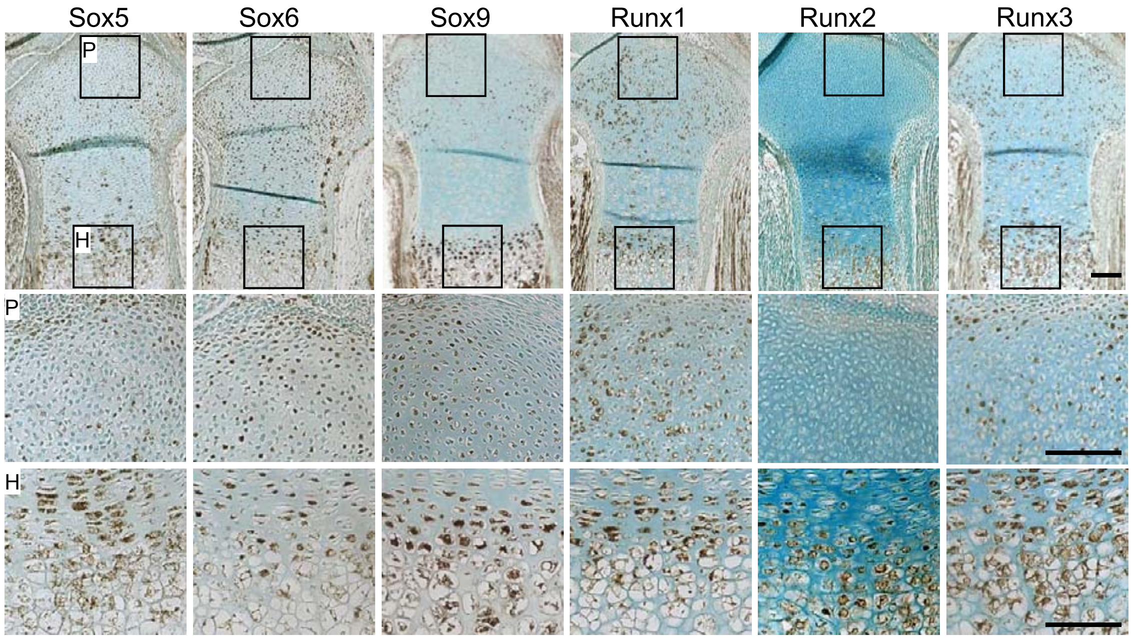

Supplementary Figure 5. Immunohistochemistry of Sox and Runx proteins in mouse tibial limb cartilage (E17.5). Inset boxes in the upper images indicate the regions of the respective lower images at higher magnification. P, periarticular zone; H, hypertrophic zone. Scale bars, 100 µm.

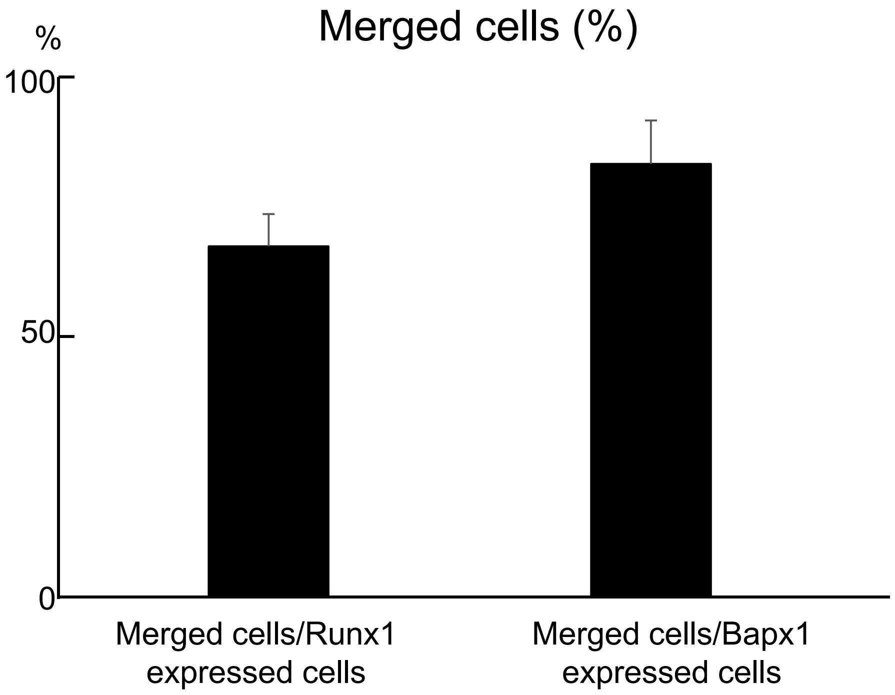

Supplementary Figure 6. Merged cells percentage (Merged cells/Runx1 expressed cells and Merged cells/Bapx1 expressed cells) are shown by Immunofluorescence with antibodies to GFP and Bapx1 in knee joints of Runx1-IRES-GFP mice under physiological condition (Figure 4b).

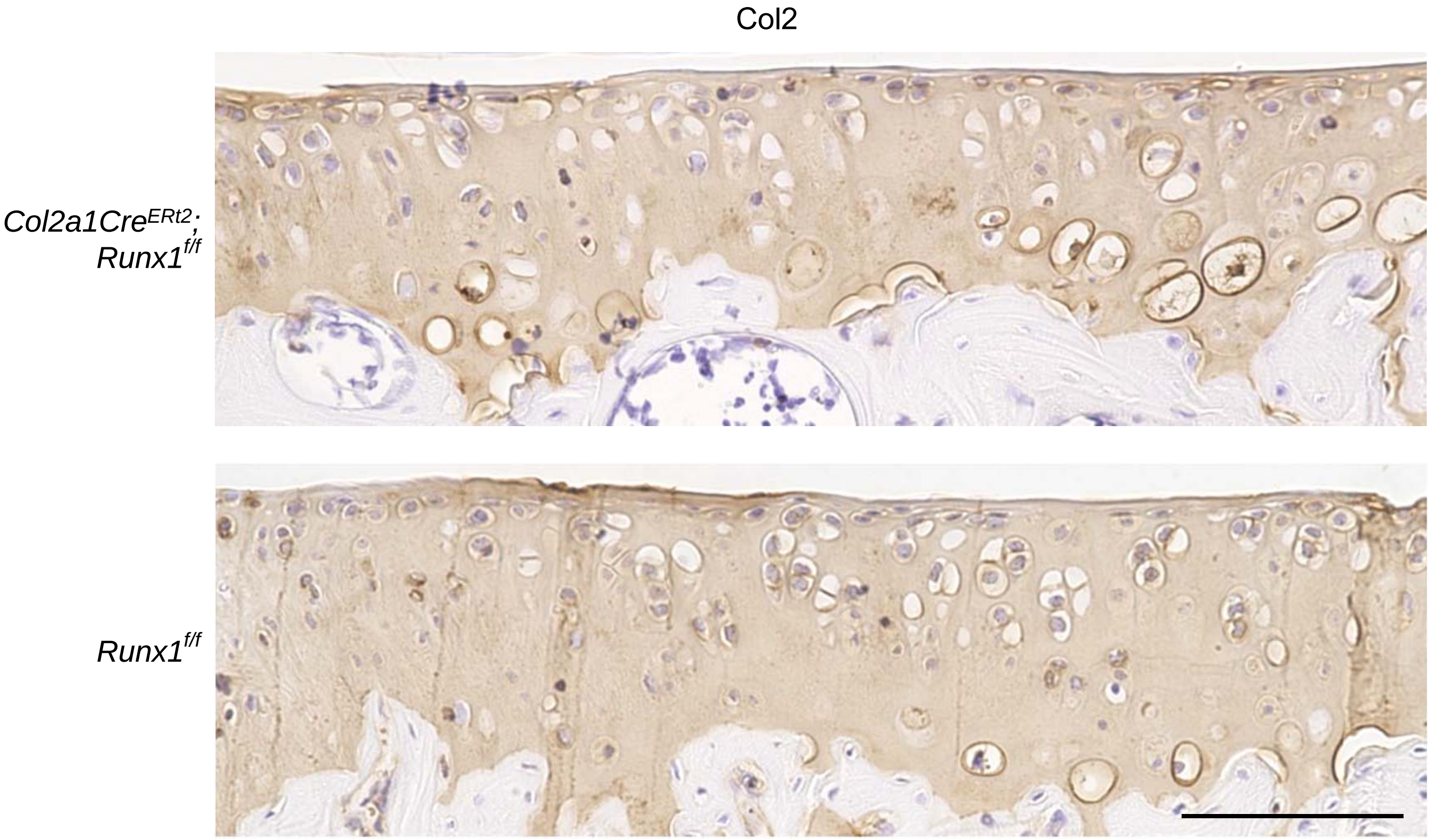

Supplementary Figure 7. Immunohistochemistry of Col2 in articular cartilage of 16-week-old Col2a1-Cre;Runx1fl/fl and Runx1fl/fl littermates under physiological conditions. Scale bars, 100 μm

qRT-PCR primers

GENES	FORWARD	REVERSE
m-Actin	AGATGTGGATCAGCAAGCAG	GCGCAAGTTAGGTTTTGTCA
m-Runx1	CAGGCAGATCCAGCCATC	TTGAGAGTCGACTGGAAAGTTCT
m-Col2a1	GCCAAGACCTGAAACTCTGC	GCCATAGCTGAAGTGGAAGC
m-Sox9	CGACTACGCTGACCATCAGA	AGACTGGTTGTTCCCAGTGC
m-Sox6	GGATTGGGGAGTACAAGCAA	CATCTGAGGTGATGGTGTGG
m-Col10a1	CATAAAGGGCCCACTTGCTA	TGGCTGATATTCCTGGTGGTA
m-Bapx1	GCGTGACGACCAAAGACAGTATTT	GGGAGACAGTAGTAAGGGTAGTAGTAGGA
